# Prevalence and nature of sexual violence in a gerontopsychiatric population in flanders

**DOI:** 10.1192/j.eurpsy.2021.380

**Published:** 2021-08-13

**Authors:** A. Nobels, I. Keygnaert, E. Robert, C. Vandeviver, N. Van Den Noortgate, G. Lemmens

**Affiliations:** 1 International Centre For Reproductive Health, Ghent University, Gent, Belgium; 2 Criminology, Criminal Law And Social Law, Ghent University, Ghent, Belgium; 3 Research Foundation—flanders, FWO, Brussels, Belgium; 4 Geriatrics, Ghent University Hospital, Ghent, Belgium; 5 Psychiatry, Ghent University Hospital, Ghent, Belgium

**Keywords:** older adults, ageing, elder abuse and neglect, old age psychiatry

## Abstract

**Introduction:**

Sexual violence (SV) is an important public health concern which may induce important and long lasting mental health problems. However, studies on SV and its mental health impact on older adults and more specifically gerontopsychiatric patients are currently lacking.

**Objectives:**

This study aims to contribute to a better understanding of the prevalence, risk factors and mental health impact of SV in a gerontopsychiatric patient population.

**Methods:**

Between July 2019 and March 2020 100 patients (66%F, 34%M) participated in a face to face interview on health, sexuality and wellbeing during their admission at an old age psychiatry ward in one general hospital and two psychiatric hospitals across Flanders, Belgium. Participation rate was 58%. Interviews were performed by a psychiatric trainee and especially trained master students in medicine.

**Results:**

58% (65%F; 42%M) of the participants were sexually victimised during their life, 45% (51%F, 33%F) experienced hands-off SV, 43% (48%F, 33%M) sexual abuse with physical contact and 16% (6%M, 21%F) was raped. 7% were sexually victimised in the past year. Compared with non-victimized respondents, hands-on SV victims (incl. rape) described more symptoms of depression (p=0.007) and anxiety (p=0.003) and reported lower resilience (p=0.022).

**Conclusions:**

SV appears to be common in the gerontopsychiatric population and is linked to even worse mental health outcomes. These findings confirm the long-lasting mental health impact of SV and highlight the importance of attention to (sexual) trauma in mental health care in old age.

**Disclosure:**

No significant relationships.

